# How much time do internal medicine residents spend on self-directed learning and on which resources: a multi-center study

**DOI:** 10.1080/10872981.2025.2501259

**Published:** 2025-05-17

**Authors:** Shreya P. Trivedi, Anthony R. Artino, Adam Rodman, R. Logan Jones, Jafar Al-Mondhiry, Timothy Rowe, Tyler Larsen, Sarai Ambert-Pompey, Devesh Rai, Ahmed Ghoneem, Nicholas Gowen, Melina Manolas, Martin Fried, Shrunjal Trivedi, Kelly L. Graham

**Affiliations:** a Division of Medicine, Beth Israel Deaconess Medical Center, Boston, MA, USA; b Shapiro Center for Education, Beth Israel Deaconess Medical Center, Boston, MA, USA; c School of Medicine and Health Sciences, George Washington University School of Medicine and Health Sciences, Washington, DC, USA; d Department of Medicine, Oregon Health and Science University, Portland, OR, USA; e Inova Schar Cancer Institute, Fairfax, VA, USA; f Department of Hematology/Oncology, University of Virginia School of Medicine, Charlottesville, VA, USA; g Division of Pulmonary and Critical Care Medicine, McGaw Medical Center of Northwestern University, Chicago, IL, USA; h Department of Medicine, VA Greater Los Angeles, Los Angeles, CA, USA; i Department of Medicine, Boise VA Medical Center, Boise, ID, USA; j Department of Cardiology, Sands-Constellation Heart Institute, Rochester Regional Health, Rochester, NY, USA; k Department of Cardiovascular Medicine, University of Pittsburgh Medical Center, Harrisburg, PA, USA; l Department of Medicine, University of Arkansas for Medical Sciences, Little Rock, AR, USA; m Department of Medicine, New York Presbyterian-Weill Cornell Medical Center, New York, NY, USA; n Department of Internal Medicine, Ohio State University Wexner Medical Center, Columbus, OH, USA

**Keywords:** Self-directed learning, clinical learning environment, graduate medical education, learning resources, technology in teaching, survey design

## Abstract

Increased clinical demands and newer means of self-directed learning (SDL) necessitate an understanding of how medical residents are supporting their learning. To examine the patterns of SDL engagement among internal medicine residents, their attitudes and behaviors with various resources, and evaluate the relationship between the clinical learning environment (CLE) and the time residents allocate to SDL and types of resources. This cross-sectional study used a systematic questionnaire informed by previous qualitative research on SDL among internal medicine residents. Internal medicine (IM) residents from 10 residency programs across the United States participated, providing a diverse representation of geographical and institutional contexts. Residents were asked to estimate weekly hours spent on SDL during their last clinical rotation, on which resources, and then to rank the usefulness of each resource. The survey also measured several variables, including attitudes and behaviors after using the resource they perceived to be the most useful, and the influence of training level, residency program type, clinical rotation, and number of hours worked clinically per week on reported time spent on SDL and types of resources. The response rate was 69.5% (783/1,126). Residents dedicated a mean of 18.2 (SD 18.6) hours per week (median of 10.5 hours per week) to SDL. Community-based programs reported more hours of SDL. There was no difference in hours spent on SDL based on the last clinical rotation, number of hours worked clinically, or PGY level. Senior residents favored digital resources, like podcasts, and were less likely to use traditional resources, like textbooks than interns. Our findings underscore the substantial time residents devote to SDL. In light of these results, educators and healthcare systems will need to work together to better support residents in optimizing the complex clinical learning environment.

## Introduction

The clinical learning environment (CLE) has changed significantly over the last two decades. Clinical demands have increased as hospitals prioritize throughput despite facing staffing shortages and having increasing clinical complexity of an aging patient population [1]. As the clinical demands change, the learning needs of resident physicians may change as well. Furthermore, the growing burden of administrative work stemming from these trends leaves less time in the day for trainees to reflect on teaching points, with 7 out of 10 residents reporting emotional exhaustion and depersonalization after an internal medicine inpatient rotation [2]. At the same time, the learning environment has evolved to offer more options for learning, with readily available videos, podcasts, and succinct teaching points on social media platforms. Amidst all of these changes in the CLE, it is essential for medical educators to understand how residents engage in self-directed learning (SDL) in order to optimize their educational environment.

Self-directed learning (SDL) refers to learning activities that trainees undertake on their own initiative. SDL allows residents to tailor their educational experience to their specific needs, areas of interest, and gaps in knowledge. The nature of residency training – with its emphasis on experiential learning – particularly encourages autonomy and responsibility for one’s own learning. Additionally, SDL fosters lifelong learning, a crucial skill as residents learn to continue their professional development beyond formal training [3]. This approach also builds essential time-management and self-assessment practice for the fast-paced and often unpredictable medical environment [4].

In the last decade, only one study evaluated the perceptions of learning resources among Internal Medicine (IM) residents. This study found that digital platforms like podcasts and streaming videos were perceived as more helpful than textbooks and residency curricula [5]. In addition, our prior qualitative work on how residents support their learning highlights the tension between the cognitive strain of the clinical environment and residents’ choices for their SDL [6]. In light of this prior work, we seek to add to the extant literature by (1) measuring in a multi-institutional cohort how residents prioritize their time for learning and with which modalities of SDL resources, (2) describing how different modalities may support different attitudes and skills, and (3) examining how aspects of the clinical learning environment may influence the total time spent on SDL and specific resource utilization.

## Materials and methods

### Setting and population

This was a cross-sectional study of internal medicine (IM) residents representing 10 residency programs from across the United States (US). Using professional networks, we recruited a convenience sample of programs, intending to attain geographic diversity from most major US regions (all regions are represented except for the Southeast). In an effort to capture diversity in the types of IM residents represented, three programs self-identified as ‘community-based’ residency programs (defined as residency programs not directly affiliated with a medical school or one in which the majority of clinical rotations do not take place at the main medical center or university hospital). None of the participating programs allotted specific protected time for independent study. The Institutional Review Board (IRB) at each participating site reviewed and approved the protocol. In all cases, the study was deemed exempt from ongoing oversight.

Between October 2021 and April 2022, we emailed all IM residents at each participating institution an electronic link of the survey (see questionnaire in Supplemental Appendix A). We sent up to two reminders to non-responders.

### Instrument design

Using a systematic process for questionnaire design [7], we constructed our main 10-item tool informed by our own qualitative work and through a review of the relevant literature [5,6,8–11]. We defined SDL as learning activities that ‘you do for yourself’ with the constraints that it was (1) not assigned to you, and (2) not completed at the point-of-care (for example, using UpToDate to look up antibiotic duration in pneumonia). To improve content validity, we consulted with 8 content and methods experts to refine the questionnaire. We also conducted 15 cognitive interviews with IM residents who were not part of the final survey sample. This convenience sample of interviews were conducted by the lead P.I and included residents from the authors’ professional networks. The purpose of the cognitive interviews was to collect response process validity and ensure that the survey questions were interpreted consistently across respondents’ diverse backgrounds [12].

### Main measures

To understand how much time residents spend on SDL and on which resources, we asked residents to estimate the number of hours per week on their last rotation they spent on various resources. We also asked them to rank the top 3 resources they perceived as most useful (with 1 being most useful). The eight resource choices provided to respondents were based on our prior qualitative research in which residents enumerated all the learning resources they used [6]. From those responses, we finalized the eight categories used in this survey to reflect the distinct types of SDL. As such, respondents were not given an open-text option to add other resources, since these predefined categories were intended to comprehensively capture the relevant learning modalities. The resource choices included reading medical textbooks, journal articles, clinical decision support tools, such as UptoDate, listening to podcasts, watching online videos, reading blogs, using social media platforms and using question banks. We also intentionally anchored the survey questions to their last rotation to improve accuracy in recalling specific resources, amount of time spent, and perception of usefulness.

For the second aim of describing how different resources may or may not support different aspects of learning, we asked respondents the extent to which they agreed or disagreed with statements of attitudes and behaviors for the resource they perceived to be the most useful. We purposefully included positive as well as negative attitudes (ex. feeling more curious or comfortable with uncertainty as well as feeling overwhelmed or burnt out) after engaging with a particular resource.

The final aim focused on the relationship between the clinical learning environment and SDL. We assessed the association of total time spent on SDL and specific resource utilization (measured in aim 1) and the following aspects of the clinical learning environment: training level, type of clinical rotation, number of hours worked clinically, and program influence (self-identified community vs. non-community residency program).

### Analysis

We calculated descriptive statistics for all the measured variables, and we present the median and mean number of hours per week spent on each type of resource. We also report the top 3 most useful resources for SDL. For resources that resident’s reported as most useful, we dichotomized the responses, grouping those who strongly agreed or agreed into the ‘agree’ category and we report those percentages.

To assess the relationship between the clinical learning environment on SDL, we evaluated differences in the reported number of hours per week spent on SDL by the type of rotation, the number of hours worked clinically, the program and PGY level using analysis of variance (ANOVA) testing, and compared means among different types of programs (self-identified ‘community’ program vs. non-community program) using T-test. We used logistic regression to assess the different clinical learning environment factors in predicting using a particular type of resource.

## Results

Of 1,126 invited IM residents across 10 programs, 783 (69.5%) responded: 281 PGY-1, 240 PGY-2, and 262 PGY-3. Table 1 presents the response rate distribution for each of the program sites. Of the programs, three self-identified as community programs (Rochester Regional Health, BI-Lahey and Boise-Idaho). About one-third of respondents had just completed a general inpatient wards rotation, followed by ICU, continuity clinic, outpatient, elective night float, emergency department, and other electives (see Appendix B for detailed distribution).

**Table 1.** Response rates by program site.

Program Sites	Respondents	Total # of Residents	Site Specific Response Rate
Arkansas	42	64	65.6%
BI-Lahey	35	47	74.5%
BIDMC	100	155	64.5%
Boise Idaho	23	33	69.7%
Cornell	95	130	73.1%
Northwestern	81	125	64.8%
Ohio (OSU)	129	177	72.9%
Oregon OHSU	77	90	85.6%
Rochester Regional Health	83	123	67.5%
UCLA	118	182	64.8%
***Total***	783	1126	**69.5%**

### Time per week on self-directed learning & specific resources

Among all residents, the average number of hours of SDL was 18.2 (SD 18.6) hours per week (median = 10.5 hours per week). Table 2 shows the breakdown of hours spent per week on the different resources. Residents reported spending the most time using clinical decision support tools, with a mean 5.2 (SD 4.7) hours per week and median of 3.5 hours per week. Podcasts, journal articles and online videos ranged from a mean of 2.1–2.3 hours per week (median = 0.5–1 hours per week). Question banks, blogs, social media platforms and medical textbooks ranged from a mean of 1.2–2.5 hours per week with a median of 0 hours per week.

**Table 2.** IM residents’ reported number of hours per week on each resource (*n* = 783).

Mean (SD) Hours Per Week	Median (IQR) Hours Per Week	Resource
5.2 (4.7)	3.5 (6.5)	Reading clinical decision support software (e.g., UpToDate, Dynamed)
2.2 (3.1)	1 (3.0)	Reading journal articles
2.1 (3.1)	1 (2.5)	Listening to podcasts
2.3 (3.6)	0.5 (3.0)	Watching online videos (e.g., YouTube)
2.5 (4.2)	0 (3.0)	Using question banks
1.4 (2.6)	0 (1.5)	Reading blogs (e.g., Life in the Fast Lane)
1.3 (2.7)	0 (1.0)	Using social media platforms (e.g., Twitter)
1.2 (2.5)	0 (1.0)	Reading medical textbooks (paper or electronic)

### Top 3 resources perceived as most useful

About half of the residents (52%, 411/783) ranked clinical decision support tools as their most useful learning resource, followed by question banks (17%, 136/783) and listening to podcasts (9.9%, 78/783) (Table 3). When considering the top three resources ranked by each resident (not just the #1 choice), the pattern remained similar. A majority of residents (84.6%) included a clinical decision support tool among their top 3 most useful resources. About half (48.4%) included question banks, and 41.3% included listening to podcasts in their top 3 most useful.

**Table 3.** IM residents’ choice for the most useful resource (%) (*n* = 783).

Reading clinical decision support tool (e.g., UpToDate, Dynamed)	411 (52.4%)
Using question banks	136 (17.3%)
Listening to podcasts	78 (9.9%)
Watching online videos (e.g., YouTube)	54 (6.8%)
Reading medical textbooks (paper or electronic)	32 (4.1%)
Reading journal articles	22 (2.8%)
Using social media platforms (e.g., Twitter)	18 (2.3%)
Reading blogs (e.g., Life in the Fast Lane)	17 (2.2%)

### Attitudes and behaviors among the resource perceived to Be most useful

**Figure 1.**
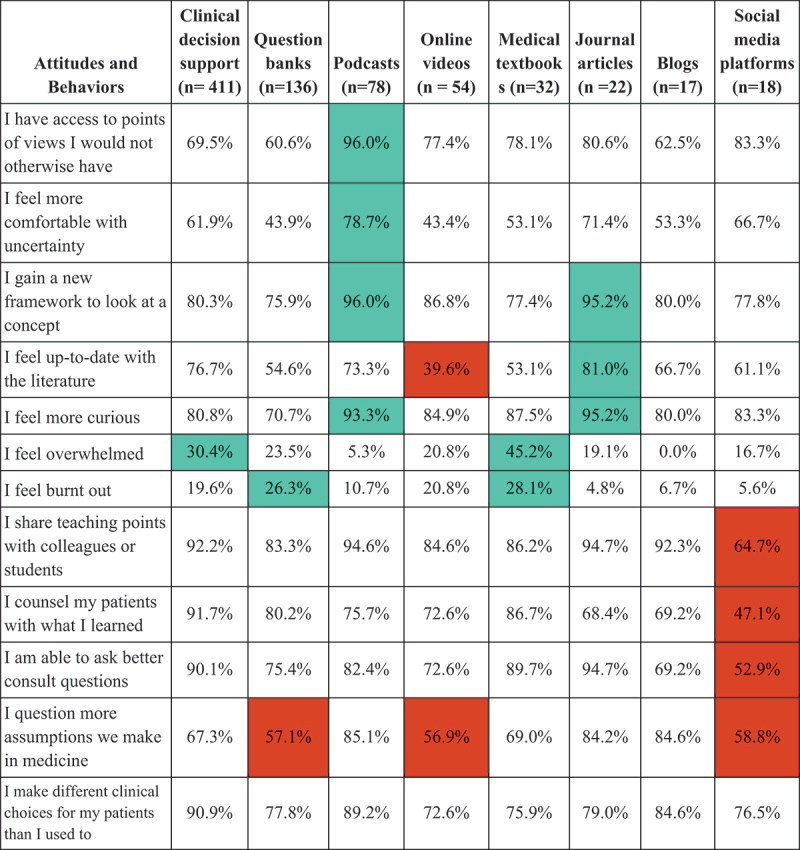
Percentage of IM residents’ that agree with attitudes and behaviors.

Note: The percentages represent the residents who agreed or strongly agreed to a statement about the resource they found to be most useful. Green shading indicates a relatively higher proportion of residents who agreed with the given statement for that resource (compared to other resources); whereas red shading indicates a relatively lower proportion of agreement. Neutral responses (‘Neither agree nor disagree’) were not counted as agreement no disagreement. The green and red color coding does not represent statistical significance; instead, it is used for illustrative purposes only to highlight patterns of agreement.

Based on the resource that each resident chose as their most useful, we then asked the extent to which they agree or disagree about several attitude and behavioral items after using that particular resource (Figure 1).

Residents who found podcasts most useful showed the strongest agreement that podcasts provided access to perspectives they would not otherwise have encountered and helped them feel comfortable with uncertainty. Furthermore, the majority of residents who valued podcasts and journal articles agreed that these resources provided new frameworks for understanding concepts and increased their curiosity.

There was most agreement for feeling overwhelmed among those who perceived textbooks and reading clinical decision support tools as most useful (45% and 30%, respectively). Feeling burnout was agreed upon more frequently among those who felt reading medical textbooks and use question banks as the most useful (28% and 26%, respectively).

### Time Spent on SDL & Clinical Learning Environment Factors

There was no significant difference among PGY-1s, PGY-2s and PGY-3s with respect to the reported time spent on SDL per week (*p* = 0.794). Similarly, there was no significant relationship between the amount of time spent on SDL and the number of hours of clinical work per week (*p* = 0.75) on their last clinical rotation or type of clinical rotation (*p* = 0.24) (See Appendix Tables 2 and 3). There was, however, a significant difference in the average amount of time on SDL between community-based programs with an average of 26.5 (SD 22.1) hours per week (median = 21.0 hours/week) and non-community-based programs with an average of 16.3 (SD 17.2) hours per week (median 10.0 hours/week). Table 4 shows the distribution of mean and median hours per week reportedly spent on SDL among individual residency programs.

**Table 4.** Reported hours spent on self-directed based on program.

	Mean (SD)	Median (IQR)	P value
**Program**			**<0.001**
Rochester Regional Health	29.6 (23.7)	24.5 (30.5)	
BI-Lahey	24.5 (18.7)	17.5 (25.5)	
Boise Idaho	18.4 (18.3)	14.5 (21.5)	
Ohio (OSU)	20.3 (18.8)	13.5 (18.0)	
Arkansas	17.8 (15.7)	12.5 (16.0)	
UCLA	17.3 (18.7)	9.8 (17.0)	
BIDMC	15.0 (15.8)	9.3 (13.5)	
Northwestern	16.3 (18.0)	9.0 (12.0)	
Oregon OHSU	13.1 (14.7)	8.0 (12.5)	
Cornell	13.2 (16.0)	7.0 (11.5)	

### Specific resource utilization & clinical learning environment factors

When comparing the specific resources among different PGY levels, PGY3s were 1.9 times more likely to use podcasts in their SDL compared to PGY1s (OR 1.9, 1.05–3.56). Conversely, PGY2s were 70% less likely to use medical textbooks compared to PGY1s (OR 0.30, 0.11–0.82), and PGY3s were 63% less likely to read medical textbooks in comparison to PGY1s (OR 0.37, 0.14–0.94).

There were no significant differences in resource utilization between residents at community and non-community sites, or those on different clinical rotations or those who reported different numbers of hours worked during their last rotation.

## Discussion

Residents in our large, multi-institutional survey reported dedicating a significant amount of time to support their SDL. Learning habits have evolved such that residents in our sample committed, on average, 18.2 hours per week to their own learning. This amount of SDL time is quite a bit more than the time reported in prior studies of IM resident study habits; one study described residents reading an average of 4.3 hours a week and another reporting about three-quarters of IM residents reading less than 7 hours per week from various resources, including online resources [8,9]. Residents in our sample do have access to an array of newer resources that were not yet in routine use during these prior studies, including podcasts, videos and social media. However, our findings do not support that the amount of time dedicated to these latest resources (mean of 1–2 hours/week with a median of 0–0.5 hours/week) fully accounts for the increased hours, indicating that the increase in engagement may reflect the increased demands of CLE as well as a response to exponential growth in research and clinical practice guidelines.

In light of these findings, we hope both medical educators and trainees will better appreciate the substantial time commitment required to develop the competencies required for independent practice in today’s complex clinical learning environment. This commitment illuminates the ever-growing challenge within residency training: the tension between increasing demands of clinical service and education [13]. The clinical environment for internists is especially challenged by the growing complexity of the interdisciplinary team, clinical status of patients, communication demands leveraged by electronic medical records and hospital demands for increasing throughput and revenue. Our findings have implications for hospital systems, as we believe it is imperative that these entities recognize and address the growing demands placed on residents, thereby facilitating a more sustainable integration of education and clinical practice.

In terms of particular resources, we found that clinician decision support tools were both the most frequently used resources and were perceived to be the most useful. This finding corroborates a prior survey study among IM residents that found clinical decision-support tools were perceived as the most helpful [5]. In practice, when physicians encounter unfamiliar or complex patient cases, they often use clinical decision support tools in real time. This process of seeking out information as questions arise during patient care is consistent with the principle of adult learning theory which posits that adults learn best when information is immediately applicable and will ultimately enhance the retention and application of knowledge [14]. The next frequently used grouping of resources included one ‘traditional’ resource, medical journals, which was clustered with two newer modalities, podcasts and online videos. This finding indicates a growing popularity among podcasts and online videos, reaching the use level of medical journals, which many would consider a ‘gold standard’ of clinical learning. What is notable in this cohort is that podcasts seem to support several desirable attitudes, including curiosity and gaining new frameworks to look at concepts that more traditional modalities such as medical journals provide without increasing burnout or discomfort with uncertainty.

Our study adds to prior studies on SDL with the inclusion of self-identified community programs. There was a significant difference with residents from self-identified community programs reporting an average of 26.5 hours/week (median = 21.0 hours/week) compared to an average of 16.3 hours/week (median = 10.0 hours/week) at non-community programs. This may reflect the pressures that International Medical Graduates (IMGs) face to stand out in fellowship matches, as IMGs are less likely to practice general medicine and more likely to specialize [15]. Community-based programs may also have less full-time faculty with paid time dedicated towards medical education and therefore, residents may spend more time to support their own learning [16]. There may also be differences in the complexity of patients and the extent of administrative tasks and care coordination at community sites compared to tertiary care hospitals that afford community sites time to spend on SDL. Future studies should try to expound on why these differences exist and if more time on SDL leads to competency for independent practice sooner or better patient outcomes.

In terms of factors favoring certain resources over others, the data indicate there may be an evolution of learning modalities across training years. Notably, PGY3s were more likely to utilize podcasts compared to PGY1s. Conversely, the use of textbooks was less in advanced residency training levels. This inclination for podcast use among more senior residents might be linked to the fact that most residents who are avid users agreed that they feel more comfortable with uncertainty and have access to viewpoints they would not have otherwise after listening to podcasts. Senior residents may be seeking to refine their clinical judgment as they lead teams and can feel more confident having access to and hearing experts navigate uncertainty with complex clinical matters and the nuances of clinician decision-making [17]. Medical educators can use this data to inform their formal curricula for senior residents and engage in more thinking out loud or now with virtual means, bringing in more experts to discuss the nuances and uncertainty of a clinical topic.

In contrast, one may expect that clinical work hours or different environments (rotations, community vs academic affiliate site) might promote differences in learning resource utilization patterns. However, our findings suggest that patterns of SDL resource utilization among IM residents are relatively stable across the learning environments and contexts analyzed in this study. In one sense, this is unsurprising, since medical learners are creatures of habit and have a tendency towards what they are most familiar with [6]. What is more, this finding aligns with research in psychology more broadly, which suggests that less familiar stimuli consume more working memory resources, and familiar stimuli create an easier foundation for building new knowledge [18]. Medical educators as well as learners can intentionally pause prior to starting a different learning environment and reflect if other learning methods may be better for their learning needs and not reflexively reach for similar resources.

This study has several important limitations. While we attempted to minimize recall bias by anchoring the questions to respondents’ last rotation, limitations related to memory recall and social desirability bias may have resulted in less than accurate time estimates. What is more, because we made use of a convenience sample, we cannot make firm statements that necessarily generalize to the population of IM residents. Future directions include understanding these learning habits with AI-driven tools in SDL once AI is more readily adopted and assess if it helps to minimize the amount of time spent on SDL.

Notwithstanding the limitations described above, this study on learning habits examines a large cohort of IM residents and achieved a robust response rate across a variety of diverse sites. Taken together, our findings underscore the significant amount of time that IM residents report dedicating to SDL. Given these findings, it is imperative that educators and healthcare systems collaborate to enhance support for residents to meet the demands of the modern healthcare setting as well as their own educational needs.

## Data Availability

The original contributions presented in the study are included in the article/supplementary material, further inquiries can be directed to the corresponding authors.

## Appendix A. Questionnaire

1. Please estimate the **# of hours per week** during your last completed rotation you spent on the following educational materials for your self-directed learning.

*[Last completed rotation excludes vacation. Examples include ICU, clinic, nights, inpatient wards, elective, ED]*

(1) Reading medical textbooks (paper or electronic)
(2) Reading journal articles
(3) Reading clinical decision support software (e.g., UpToDate, Dynamed)
(4) Listening to podcasts
(5) Watching online videos (e.g., YouTube, recorded lectures)
(6) Reading blogs (e.g., Life in the Fast Lane)
(7) Using social media platforms (e.g., Twitter)
(8) Using question banks

(2) Please write 1, 2 and 3 next your top 3 educational resources that you find **most useful** for your self-directed learning, where **1 = most useful**

(1) Reading medical textbooks (paper or electronic)
(2) Reading journal articles
(3) Reading clinical decision support software (e.g., UpToDate, Dynamed, Epocrates)
(4) Listening to podcasts
(5) Watching online videos (e.g., YouTube, recorded lectures)
(6) Reading blogs (e.g., Life in the Fast Lane)
(7) Using social media platforms (e.g., Twitter)
(8) Using question banks

(3) When using **[PIPE TOP RESOURCE FROM Q2**], please indicate the extent you agree or disagree with the following:

**[STRONGLY AGREE - SOMEWHAT AGREE -NEUTRAL - SOMEWHAT DISAGREE - STRONGLY DISAGREE RESPONSE OPTIONS]**

(1) I feel more curious
(2) I feel overwhelmed
(3) I gain a new framework to look at a concept
(4) I feel up-to-date with the literature
(5) I have access to points of views i would not otherwise have
(6) I feel **more** comfortable with uncertainty
(7) I feel more connected to peers and/or faculty
(8) I feel burnt out

(4) After using **[PIPE TOP RESOURCE FROM Q2]** please indicate the extent you agree or disagree with the following:

**[STRONGLY AGREE - SOMEWHAT AGREE -NEUTRAL - SOMEWHAT DISAGREE - STRONGLY DISAGREE RESPONSE OPTIONS]**

(1) I share teaching points with colleagues or medical students
(2) I counsel my patients with the information I learned
(3) I am able to ask better consult questions
(4) I question more assumptions we make in medicine
(5) I make different clinical choices for my patients than I used to
(6) I start projects based on ideas introduced from this resource

(5) After using **[PIPE TOP RESOURCE FROM Q2]**, how often do you… (matrix: Read a cited abstract; Read a cited paper)
  (1) Almost always
  (2) Most of the time
  (3) Sometimes
  (4) Rarely
  (5) Almost never

(6) **[BRANCH: For those who answer sometimes, most of the time, or always]** what motivates you to look at cited reference when using [PIPE TOP RESOURCE FROM Q2]s? [text limits]

(7) After using **didactics** (e.g noon conference or morning rounds), how often do you: (matrix: Read a cited abstract; Read a cited paper)
  (1) Almost always
  (2) Most of the time
  (3) Sometimes
  (4) Rarely
  (5) Almost never

(8) What was your **last completed rotation** (excluding vacation)?
  (1) Intensive care unit
  (2) Nights
  (3) Clinic
  (4) Inpatient wards
  (5) Elective - Outpatient
  (6) Elective - Inpatient
  (7) Elective - Non-clinical
  (8) Emergency Department

(9) What is the **average # of hours per week** you worked clinically in your last completed rotation?

(10) What is your level of training?
  (1) Preliminary PGY1
  (2) IM PGY-1
  (3) PGY2
  (4) PGY3
  (5) PGY4 or greater

## Appendix B. Reported hours per week based on last rotation

**Table:**

	Mean (SD) Hours Per Week	Median (IQR) Hours Per Week	P value
Last Rotation (n = No. of Residents)			0.242
Emergency Department (n=26)	22.6 (23.2)	18.8 (27.0)	
Nights (n=64)	19.0 (18.0)	12.0 (21.3)	
Elective, Outpatient (n=81)	19.2 (17.7)	11.5 (21.5)	
Clinic Rotation (n=103)	20.1 (20.7)	11.0 (20.5)	
Elective, Inpatient (n=43)	15.6 (14.8)	10.0 (10.0)	
Intensive Care Unit (n=126)	18.7 (19.0)	10.8 (18.0)	
Inpatient Wards (n=246)	16.0 (17.2)	9.5 (16.0)	
Elective, Non-clinic (n=14)	24.3 (26.0)	8.0 (44.5)	

## Appendix C. Reported hours per week spent on SDL based on hours worked clinically in last rotation

**Table:**

	Mean (SD) Hours Per Week	Median (IQR) Hours Per Week	P value
Average Hours Per Week Worked Clinically			0.242
0-20 hours	22.6 (25.9)	9.75 (29.5)	
21-40 hours	17.5 (16.9)	10.5 (19.0)	
41-60 hours	18.0 (18.6)	10.5 (17.0)	
61-80 hours	17.0 (17.7)	10.0 (17.0)	
80+ hours	15.5 (16.8)	8.5 (19.3)	
